# Predicting Response to Group Cognitive Behavioral Therapy in Asthma by a Small Number of Abnormal Resting-State Functional Connections

**DOI:** 10.3389/fnins.2020.575771

**Published:** 2020-11-24

**Authors:** Yuqun Zhang, Kai Ma, Yuan Yang, Yingying Yin, Zhenghua Hou, Daoqiang Zhang, Yonggui Yuan

**Affiliations:** ^1^School of Nursing, Nanjing University of Chinese Medicine, Nanjing, China; ^2^Department of Psychosomatics and Psychiatry, Zhongda Hospital, School of Medicine, Southeast University, Nanjing, China; ^3^MIIT Key Laboratory of Pattern Analysis and Machine Intelligence, College of Computer Science and Technology, Nanjing University of Aeronautics and Astronautics, Nanjing, China; ^4^Department of Respiratory, Zhongda Hospital, School of Medicine, Southeast University, Nanjing, China

**Keywords:** asthma, group cognitive behavioral therapy, machine learning, support vector machine, therapeutic mechanism

## Abstract

Group cognitive behavioral therapy (GCBT) is a successful psychotherapy for asthma. However, response varies considerably among individuals, and identifying biomarkers of GCBT has been challenging. Thus, the aim of this study was to predict an individual’s potential response by using machine learning algorithms and functional connectivity (FC) and to improve the personalized treatment of GCBT. We use the lasso method to make the feature selection in the functional connections between brain regions, and we utilize *t*-test method to test the significant difference of these selected features. The feature selections are performed between controls (size = 20) and pre-GCBT patients (size = 20), pre-GCBT patients (size = 10) and post-GCBT patients (size = 10), and post-GCBT patients (size = 10) and controls (size = 10). Depending on these features, support vector classification was used to classify controls and pre- and post-GCBT patients. Pearson correlation analysis was employed to analyze the associations between clinical symptoms and the selected discriminated FCs in post-GCBT patients. At last, linear support vector regression was applied to predict the therapeutic effect of GCBT. After feature selection and significant analysis, five discriminated FC regarding neuroimaging biomarkers of GCBT were discovered, which are also correlated with clinical symptoms. Using these discriminated functional connections, we could accurately classify the patients before and after GCBT (classification accuracy, 80%) and predict the therapeutic effect of GCBT in asthma (predicted accuracy, 67.8%). The findings in this study would provide a novel sight toward GCBT response prediction and further confirm neural underpinnings of asthma. Moreover, our findings had clinical implications for personalized treatment by identifying asthmatic patients who will be appropriate for GCBT.

**Clinical Trial Registration:**

The brain mechanisms of group cognitive behavioral therapy to improve the symptoms of asthma (Registration number: Chi-CTR-15007442, http://www.chictr.org.cn/index.aspx).

## Introduction

Asthma is a common respiratory disease with the clinical symptom of reversible airflow restriction, which is associated with significantly higher comorbidities including psychiatric diseases (Su et al., 2016; Shen et al., 2017). Meantime, psychiatric comorbidities have impacts on asthma management and prognosis, as they are involved in inadequate disease control and poor quality of life (Adeyeye et al., 2017; Gonzalez-Freire and Vazquez, 2017; Hekking et al., 2018). Thus, in order to clarify the underlying neural mechanisms of asthma, as well as better understand the mechanisms of psychiatric comorbidities in asthma, clinical scientists have paid more attention to asthma by using various methods (Wang et al., 2014; Xiong et al., 2016).

Cognitive behavioral therapy (CBT) is a widely used psychotherapy of exploring individuals’ perceptions and how the behavior influences their feelings and thoughts. CBT is aimed at empowering a person to change previous unhelpful thinking and behavior patterns and at developing a more positive way of thinking to acquire a more helpful behavioral response (Kew et al., 2016). Previous studies consistently demonstrated that CBT could be utilized to encourage asthmatic patients to keep control of their symptoms, accept their problems, and alleviate asthma-related negative emotions (Parry et al., 2012; Feldman et al., 2016; Kew et al., 2016). Regarding CBT in asthma including individual and group models, group CBT (GCBT) may cost less as well as acquire more social support offered by group compared with individual therapy (Yorke et al., 2017). Therefore, we applied GCBT to our study.

In recent decades, magnetic resonance imaging (MRI) shows the advancement and provides the opportunities to investigate the neural underpinnings of GCBT non-invasively, such as GCBT in depression (Sosic-Vasic et al., 2017; Sambataro et al., 2018) and anxiety (Whitfield-Gabrieli et al., 2016; Klumpp et al., 2017; MinlanYuan, Meng et al., 2017). Meanwhile, some neurophysiological mechanisms underlying GCBT for asthma have been detected in our previous studies (Zhang et al., 2017a, b), and we found that abnormal spontaneous activity and insula functional connectivity (FC) would be reversed after treatment. Emotional disorder-related studies suggested that neuroimaging biomarkers could be used in predicting response to GCBT (Doehrmann et al., 2013; Whitfield-Gabrieli et al., 2016). However, despite the significance of GCBT, no effective biomarkers have been developed. In addition, although the above neuroimaging findings have made substantial progress in demonstrating the neural mechanisms of GCBT, they are based on conventional group analyses (Hahn et al., 2015). A useful neuroimaging biomarker with sufficient sensitivity and specificity on the individual level is deficient.

Actually, neurocircuits seem to play an important underlying role in asthma. Rosenkranz et al. (2005, 2012) and Rosenkranz and Davidson (2009) reported that neural circuitry underlying the interaction between emotion and asthma symptoms, and the abnormal brain functions related to emotions, may be the neurophenotypes of asthma. Moreover, they also used PET to explore the neural mechanisms of asthma, and they found that greater activity in the mid-insula and perigenual anterior cingulate seems to reflect greater reactivity and was associated with greater airway inflammation and a more robust alpha amylase response (Rosenkranz et al., 2016). In addition, another author in their team summarized the linkages between brain and asthma, suggesting that specific circuits in the brain [e.g., anterior cingulate cortex (ACC) and insula] are activated in the relationship and intensity to the development of a late-phase response to inhaled antigen (Busse, 2012). And thus, these brain signals are predictive and associated with the development of airway inflammation as measured by sputum eosinophils. Thus, exploring the biomarkers of GCBT on the basis of abnormal brain functions in asthma would provide a novel inspiration.

It is worth noting that the novel approach based on machine learning (ML) brings prospect for the personalized treatment. According to the neuroimaging features based on ML analysis, physicians can directly make clinical decisions (Hahn et al., 2015). Moreover, ML has been widely used to predict CBT response in psychiatric disorders, particularly the combination with FC (Hahn et al., 2015; Mansson et al., 2015; Juarascio et al., 2018; Reggente et al., 2018). For example, in Reggente et al. (2018), FC was used to predict individual’s symptom severity of obsessive-compulsive disorder (OCD) after treatment with the method of ML. They found that FC within visual and default mode network before treatment significantly predicted post-treatment OCD severity. To our knowledge, no study has documented biomarkers and predicted response of GCBT in asthma on the basis of data-driven ML approach.

To address these issues, we aimed to discover brain FC associated with GCBT, which could be regarded as the discriminated biomarkers. These biomarkers could effectively classify normal controls and asthmatic patients using ML methods. In addition to classification, we also expected that these neuroimaging biomarkers could help us predict individual response to GCBT in asthmatic patients, thereby providing more clinical-related information.

## Materials and Methods

### Participants

Forty-two patients with a diagnosis of bronchial asthma without acute attacks and 42 healthy controls (HCs) were recruited. All participants underwent a functional MRI (fMRI) scan at baseline. In addition, 17 out of 42 patients completed GCBT treatment, and they received clinical assessments and fMRI scan again after 8-week treatment. One of them was eliminated due to data quality. The contents of GCBT were reported in our previous study, which consisted of eight sessions (Zhang et al., 2017a). This study was approved by the recommendations of the ethics committee (Zhongda Hospital, Southeast University, Nanjing, China, No. 2016ZDSYLL004.0) with written informed consent from all subjects. All procedures performed in studies involving human participants were in accordance with the ethical standards of the institutional research committee and with the 1964 Declaration of Helsinki and its later amendments. The clinical trial registration number is Chi-CTR-15007442.

Participants were all right-handed with education of more than 6 years. Their age ranged from 18 to 65 years. There are no electronic and metal equipment in their body (such as stent, defibrillator, and cardiac pacemaker). Participants were excluded if they (1) suffered from other respiratory diseases; (2) had a history of organic cardio, hepatic, renal, and brain disorders and abnormality; (3) have mental disorders, or drug and alcohol dependence; and (4) are pregnant or lactating.

### Clinical Assessments

Patients also received a series of clinical assessments, including 17-item Hamilton depression rating scale (HAMD) (Hamilton, 1960), Chinese version of Short Health Anxiety Inventory (CSHAI) (Zhang et al., 2015), and asthma control test (ACT) (Larsson et al., 2018). HAMD is used to assess the depressive severity at baseline and after treatment. ACT is a five-item self-rating scale, and it is used to assess the asthma control level. The cutoff score of ACT is 20, and uncontrolled asthma is recognized while the total score is less than 20. CSHAI is also a self-rating scale associated with health anxiety, and it has 18 items; 15 is the cutoff score in Chinese population.

### fMRI Data Acquisition and Preprocessing

MRI studies were performed on a 3-Tesla Scanner (Siemens, Erlangen, Germany) using a homogeneous birdcage head coil. The resting images were obtained using a gradient-recalled echo-planar imaging (EPI) pulse sequence. For each data volume, we acquire 36 continuous axial slices in descending order with 3.75-mm × 3.75-mm in-plane resolution parallel to the anterior commissure–posterior commissure line, 3-mm slice thickness, and a 0-mm gap using resting-state imaging [repetition time (TR) = 2,000 ms, echo time (TE) = 25 ms, flip angle = 90°, acquisition matrix = 64 × 64, field of view = 240 × 240 mm]. Participants lay supine with the head snugly fixed by a belt and foam pads to minimize head motion, and they were required to keep their eyes closed, to be awake, and to not think of specific things during scanning. The fMRI data were acquired over a period of 8 min 6 s.

DPABI was used to preprocess the resting-state fMRI data (Yan et al., 2016). After the first 10 time points were removed, the remaining 230 times points were corrected for timing differences between slices and for motion effects (six-parameter rigid body) using a reference volume in the center of the run. The resulting images were spatially normalized into a standard stereotaxic space using a 12-parameter affine approach and an EPI template image that was resampled to 3-mm × 3-mm × 3-mm voxels. Afterward, Friston 24 motion parameters, white matter (WM), and cerebrospinal fluid signals were regressed. The images were smoothed with a 4-mm full-width half-maximum Gaussian kernel and filtered from 0.01 to 0.08 Hz. All frames of all participants had less head motion of more than 2.0 mm of maximum displacement in any direction (*x*, *y*, or *z*) or 2.0° of angular motion. The data that have been used are confidential. We have modified the manuscript, please see the revised manuscript.

### Construction of Brain FC Network

The gray matter consists of 116 regions according to AAL atlas (Zhuo et al., 2018). Pearson correlation was calculated between regional mean time series of paired connectivity to acquire function brain networks, in which each region is regarded as a node and the connectivity of paired regions is seen as an edge (Rubinov and Sporns, 2010).

### Feature Selection

In function brain networks, there are 116 × 116 functional connectional values, which is a high-dimensional problem. Hence, the feature selection must be used in these functional connections in order to find which brain regions could be affected by asthma. Finding which brain regions or brain FC affected by asthma is more meaningful in doing classifications between the asthma patients and HCs and detecting the disease mechanisms. The embedding feature selection method that embeds the feature selection model into a classification model is a good surrogate of traditional feature selection method, such as wrapper and chi-square test (Satorra and Bentler, 2010). It can select a feature subset in a single optimization and take full advantages of the label information. Lasso (Bien et al., 2013) is a supervised model composed of a least-squared loss and L1-norm regularization term, which can obtain a sparse coefficient vector with each entry corresponding to the importance of a certain feature to the classification task, and thus, this coefficient vector can be used for feature selection. The formulation of lasso model is stated as follows:

(1) $$w=\underset{w}{\mathrm{argmin}}\sum_{i=1}^{N}∥y_{i}-w^{T}x_{i}{∥}^{2}+\lambda ∥w{∥}_{1}$$

Where ∥*w*∥_1_ denotes the L1-norm, and ∥*w*_1_∥ =|*w*_1_| + |*w*_2_|+⋯ +|*w*_*N*_|. In our research, we focus on the classification task. The linear regression could not be directly used for classification. We need to use the logistic regression to replace the linear regression. Then, we get the following formula:

(2) $$w=\mathrm{argmin}\sum_{i=1}^{N}∥y_{i}-y{∥}^{2}+\lambda ∥w{∥}_{1}$$

Where y = $\frac{y}{1+e^{-W^{T}x_{i}}}$. The *x*_*i*_ are the FCs (i.e., the edges in brain network). *y*_*i*_ are the labels (i.e., *y*_*i*_ =0 or 1) of patients and normal controls. *W* is the parameter that needs to be learned in feature selection.

**FIGURE 1:**
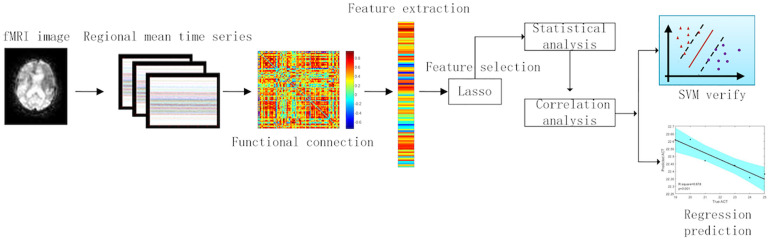
Data analysis process.

In the feature selection between patients and controls, we used 20 patients and 20 controls. We divided these 40 subjects into training set and test set with leave-one-out cross-validation (39 subjects entered into training test and 1 subject entered into test). In training set, the feature selection method is exhibited in formula (2). We tested the model learned from the training set in test data. After all, subjects have been used for test set. The FCs that always existed in each cross-validation were picked out. We applied these FCs to pick out in feature selection between 20 pre-GCBT patients and 20 controls to the statistical analysis. Furthermore, feature selection between pre- and post-GCBT patients was conducted similarly to the above method. We employed 10 pre-GCBT patients and 10 post-GCBT patients to select FC features. After related discriminated features with lasso method were acquired, we utilized the statistical (*t*-test) and correlation (Pearson correlation) analysis to detect the clinical and neural significance in these discriminated features between 20 pre-GCBT patients and 20 controls. Therefore, we can discover more representative biomarkers in brain regions and functional connections. The analysis process could be seen in Figure 1.

### Validation of Classification

In this experimental setting, we used the FCs picked out with statistical analysis to classify pre- and post-GCBT patients, as well as controls. We adopt non-linear classification method based on support vector classification (SVC) with radial basis function (RBF) kernel to classify the other 22 patients without GCBT and 22 controls. We trained the mode on the discriminated features from statistical analysis among 40 subjects including 20 pre-GCBT patients and 20 controls. We test the discriminated features on the other 44 subjects including 22 patients without GCBT and 22 controls. The discriminated FCs are also used to predict clinical information. In the feature selection between pre- and post-GCBT patients, we used 10 pre- and post-patients, respectively. Moreover, feature selection between controls and post-GCBT patients was made with 10 controls and 10 post-GCBT patients. Then, *t*-test is used to investigate the statistical differences and pick out the discriminated FCs. Depending on these discriminated features, SVC was used to classify pre- and post-GCBT patients. In the classification between pre- and post-GCBT, six pre- and post-GCBT patients were employed, respectively. Moreover, six post-GCBT patients and six controls were entered into the classification between post-GCBT patients and controls.

The parameters C (parameter of slack variable) and g (parameter of RBF kernel) in SVC were tuned from 10^–3^ to 10^3^. These parameters are tuned within training data by leave-one-out cross-validation. We choose the features that frequently occur in each cross-validation process for the statistical analysis. For example, FC between the right medial superior frontal gyrus (SFGmed) and right superior cerebellum always occurs in each cross-validation. This FC is applied to statistical analysis.

The classifier adopted in our experiments was SVC, which is the most popular classification model for its stability and power (Wang et al., 2016). SVC is formulated as follows:

(3) $$\min_{\text{w},\text{b}}\frac{1}{2}{\text{w}}^{2}+\text{C}\sum_{\text{i}=1}^{\text{N}}{\text{ξ}}_{\text{i}}$$

$$\text{s}.\text{t}.y_{\text{i}}\left({\text{w}}^{\text{T}}{\text{x}}_{\text{i}}+\text{b}\right)\geq 1-{\text{ξ}}_{\text{i}},\xi_{\text{i}}\geq 0,i=1,2,\cdots ,\text{N}$$

where _*w*_ denotes the coefficient vector that corresponds to a discriminative hyperplane with a bias _*b*_. *x*_*i*_ denotes the feature of one sample (patient or health control) with its label *y*_i_. *C* denotes the trading-off parameter for slack variable *ξ*_i_. By maximizing the minimum margin between two classes, SVC can obtain comparable performance in accuracy, generalization, and robustness.

### Regression Prediction and Correlation Analysis

We adopted the support vector regression (SVR) to predict individual response to GCBT in asthmatic patients. GCBT patients (size = 16) are divided into train sets (size = 10) and test sets (size = 6). SVR is formulated as follows:

(4) $$\min_{\text{w},\text{b}}\frac{1}{2}{\text{w}}^{2}+\text{C}\sum_{\text{i}=1}^{\text{N}}\left({\text{ξ}}_{\text{i}}+\hat{{\text{ξ}}_{\text{i}}}\right)$$

$$\text{s}.\text{t}.f\left({\text{x}}_{\text{i}}\right)-y_{i}\leq +{\text{ξ}}_{\text{i}},$$

$$y_{i}-\text{f}\left({\text{x}}_{\text{i}}\right)\leq +{\text{ξ}}_{\text{i}},$$

$${\text{ξ}}_{\text{i}}\geq 0,\hat{{\text{ξ}}_{\text{i}}}\geq 0i=1,2,\cdots ,\text{N}$$

where _*w*_ denotes the coefficient vector that corresponds to a discriminative hyperplane with a bias _*b*_. *x*_*i*_ denotes the feature of one sample (patient with GCBT) with its label *y*_i_. *C* denotes the trading-off parameter for slack variable *ξ*_i_ and $\hat{\text{ξ}{}_{\text{i}}}$.

The *R*^2^ between the predicted clinical symptoms and the actual clinical symptoms was calculated. The Pearson correlation was applied to calculate the relationships between scale scores and the discriminated functional connections in patients after treatment. And we also used correlation analysis to find the relationships between the changes of functional connections and the changes of clinical scale scores from pre- and post-treatment patients.

### Statistical Analysis

We employed Predictive Analytic Software (PASW) Statistics 18 (IBM Corporation, Armonk, NY, United States) to complete the statistical analyses. Comparisons of continuous variables (e.g., age, education, and clinical symptoms) were analyzed with two-sample *t*-test and paired *t*-test. Chi-square test was used to compare the classified variable (e.g., gender). *P* < 0.05 was considered to indicate statistical significance. The discriminated functional connections were compared between controls and patients at baseline with multiple comparisons (Bonferroni correction), as well as between controls and post-GCBT patients (uncorrected) based on two-sample *t*-test. Paired *t*-test was used to compare the connections between pre-GCBT and post-GCBT patients (uncorrected).

## Results

### Demographic and Clinical Data

Table 1 shows the detailed demographic and clinical information. No significant differences between asthmatic patients and HCs in age, gender, and education level were found in this study. Compared with controls, patients have significantly higher HAMD scores (*P* < 0.001). After GCBT, the CSHAI scores (*P* < 0.05) and HAMD scores (*P* < 0.001) of patients were lower than before. Although there were no statistical differences between the pre- and post-GCBT groups, the ACT scores had a trend of increase after treatment (*P* = 0.073).

**TABLE 1:** The demographics and scale scores of subjects.

Characteristics		Asthma (*N* = 42)	HCs (*N* = 42)	*P-*value
Age (years)		51.88 ± 9.96	50.31 ± 11.75	0.51
Gender (male)		18	17	0.825^*a*^
Education (years)		11.81 ± 2.58	11.31 ± 3.40	0.50
Duration of asthma (years)		22.04 ± 19.44	NA	
ACT		17.62 ± 4.86	NA	
CSHAI	Total scores	13.26 ± 6.51	NA	
	IL	10.26 ± 5.49	NA	
	NC	2.81 ± 2.29	NA	
HAMD		6.00 ± 5.37	1.05 ± 1.43	<0.001
		Pre-GCBT (*N* = 16)	Post-GCBT (*N* = 16)	*P-*value
ACT		16.375 ± 4.40	20.88 ± 4.35	0.073
CSHAI	Total scores	13.88 ± 7.77	12.56 ± 5.63	0.030
	IL	11.38 ± 6.02	10.44 ± 4.83	0.049
	NC	2.50 ± 2.50	2.13 ± 1.63	0.025
HAMD		5.94 ± 5.37	1.94 ± 2.38	<0.001

*Data are expressed as mean ± standard deviation.HCs, healthy controls; ACT, asthma control test; CSHAI, Chinese version of Short Health Anxiety Inventory; IL, illness likelihood (a factor of CSHAI); NC, negative consequence (a factor of CSHAI); HAMD, Hamilton depression rating scale. ^a^Chi-square test.*

### Discriminated Functional Connections

**FIGURE 2:**
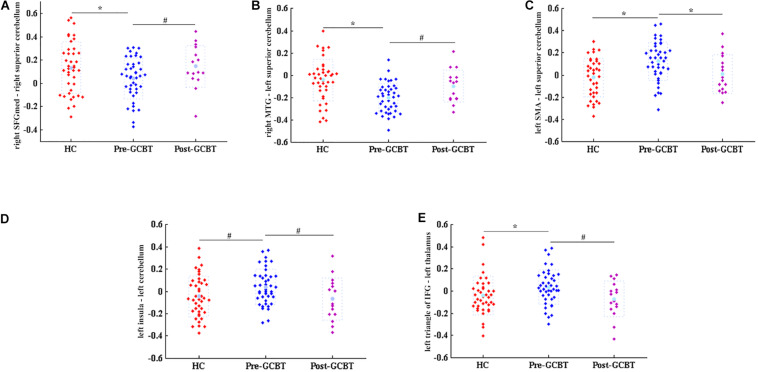
Discriminated functional connections. These five discriminated functional connections would be regarded as neuroimaging biomarkers of GCBT (*P < 0.05, Bonferroni correction; ^#^*P* < 0.05, uncorrected). **(A)** The comparisons of FC values between right SFGmed and right superior cerebellum among three groups. This FC value of asthmatic patients was significantly lower than that of HCs. After GCBT, this FC value was increased compared with that pre-GCBT and has no statistical difference compared with that of HCs. **(B–E)** Similar to **(A)**; they all showed the same variation tendency. HCs, healthy controls; GCBT, group cognitive behavioral therapy; SFGmed, medial superior frontal gyrus; MTG, middle temporal gyrus; SMA, supplementary motor area; IFG, inferior frontal gyrus.

We used the lasso method to make the feature selection in the functional connections between brain regions, and then the *t*-test was utilized to test the significant difference of these selected features. We find out five discriminated FCs that were abnormal in asthma, but there were no statistical differences between HCs and post-GCBT patients (Table 2). They are the FCs between the right SFGmed and right superior cerebellum, right middle temporal gyrus (MTG) and left superior cerebellum, left supplementary motor area (SMA) and left superior cerebellum, left insula and left superior cerebellum, and left triangle of inferior frontal gyrus (IFG) and left thalamus. Figure 2 shows that the FC values of patients after treatment were reversed and more close to the values of HCs.

**TABLE 2:** Five discriminated functional connections.

Number	Regions	Regions	*P*
1	Right medial superior frontal gyrus	Right superior cerebellum	0.023
2	Right middle temporal gyrus	Left superior cerebellum	0.042
3	Left supplementary motor area	Left superior cerebellum	0.001
4	Left insula	Left cerebellum	0.034
5	Left triangle of inferior frontal gyrus	Left thalamus	0.021

### FC as GCBT’s Biomarkers

**FIGURE 3:**
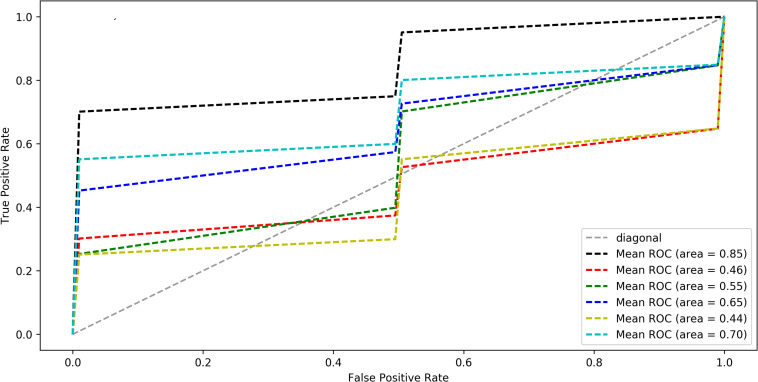
The average ROC curve under leave-one-out cross-validation. The black line was the ROC of five discriminated functional connections, the red line was first FC, the green line was second FC, the blue line was third FC, the yellow was fourth FC, and the cyan line was fifth FC (five FCs are shown in Table 2). ROC analysis differentiate post-GCBT patients from those pre-treatment patients by using five connections. The area under ROC curve was 0.85 (*P* < 0.01). ROC, receiver operating characteristic; FC, functional connectivity.

In order to further verify whether these discriminant features would be regarded as neuroimaging biomarkers of GCBT to discriminate the pre- and post-GCBT patients, we utilized the ML method based on SVC to classify these patients. The classification accuracy was 80%, and the area under the receiver operating characteristic (ROC) curve was 0.85 (specificity = 80.1%, sensitivity = 79.3%) depending on these discriminant features (see Figure 3).

### Correlations Between Connections and Clinical Symptoms

**FIGURE 4:**
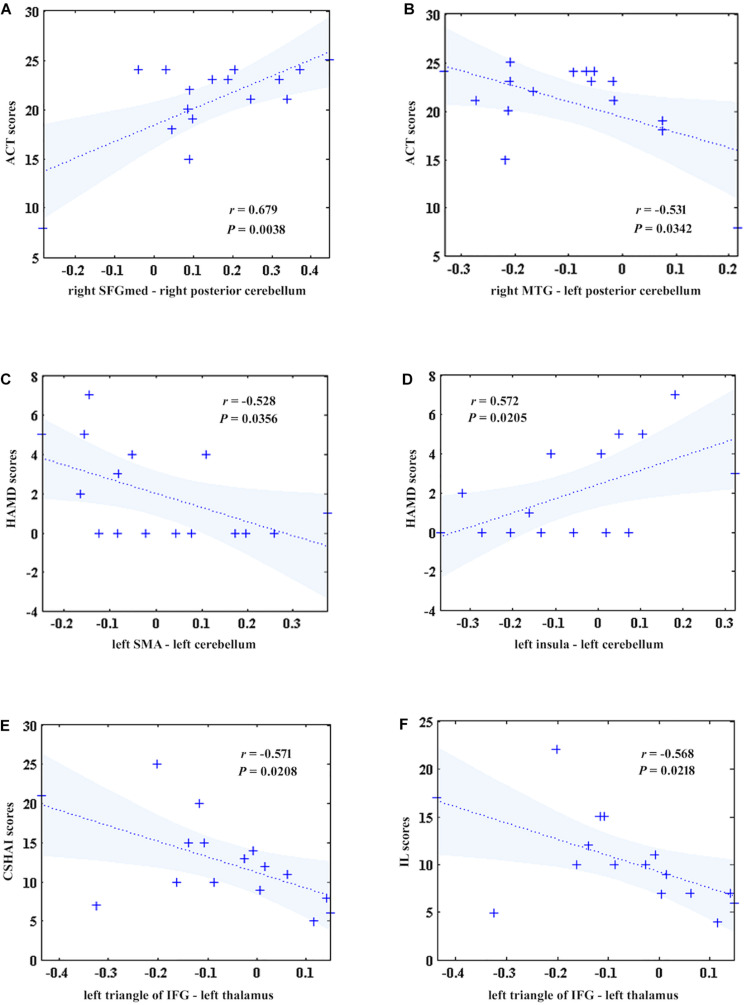
The correlations between five discriminant features and clinical symptoms in patients after treatment. **(A)** The FC between the right SFGmed and right superior cerebellum was positively correlated with ACT scores (*r* = 0.679, *P* = 0.0038). **(B)** The FC between the right MTG and left superior cerebellum was negatively correlated with ACT scores (*r* = –0.531, *P* = 0.0342). **(C)** The FC between the left SMA and left superior cerebellum was negatively correlated with HAMD scores (*r* = –0.528, *P* = 0.0356). **(D)** The FC between the left insula and left superior cerebellum was positively correlated with HAMD scores (*r* = 0.572, *P* = 0.0205). **(E)** The FC between the left IFGmed and left thalamus was negatively correlated with CSHAI scores (*r* = –0.571, *P* = 0.0208). **(F)**. The FC between the left IFGmed and left thalamus was negatively correlated with IL scores (*r* = –0.568, *P* = 0.0218). ACT, asthma control test; SFGmed, medial superior frontal gyrus; MTG, middle temporal gyrus; IFG, inferior frontal gyrus; HAMD, Hamilton depression rating scale; SMA, supplementary motor area; CSHAI, Chinese version of Short Health Anxiety Inventory; IL, illness likelihood (a factor of CSHAI).

To investigate the correlations between the FC and clinical symptoms of patients after GCBT, Pearson correlation analysis were used to detect the correlations between the FCs and clinical symptoms of post-GCBT patients. Both FCs between the right SFGmed and right superior cerebellum (*r* = 0.679, *P* = 0.0038, Figure 4A), right MTG, and left superior cerebellum (*r* = −0.531, *P* = 0.0342, Figure 4B) showed significant correlations with ACT scores. And both FCs between the left SMA and left superior cerebellum (*r* = −0.528, *P* = 0.0356, Figure 4C), left insula, and left superior cerebellum (*r* = 0.572, *P* = 0.0205, Figure 4D) showed significant correlations with HAMD scores. Moreover, FC between the left triangle of IFG and left thalamus was negatively associated with both CSHAI scores (*r* = −0.571, *P* = 0.0208, Figure 4E) and IL scores (*r* = −0.568, *P* = 0.0218, Figure 4F).

**FIGURE 5:**
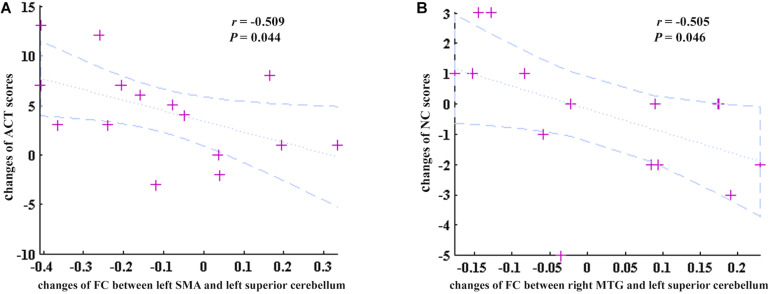
The correlations between the changes of discriminate FC features and changes of clinical symptoms. **(A)** The changes of FC between the left SMA and left superior cerebellum were negatively correlated with the changes of ACT scores (*r* = –0.509, *P* = 0.044). **(B)** The changes of FC between the right MTG and left superior cerebellum were negatively correlated with the changes of NC scores (*r* = –0.505, *P* = 0.046). ACT, asthma control test; SMA, supplementary motor area; NC, negative consequences (a factor of CSHAI); MTG, middle temporal gyrus.

In addition, the changes of FC values between the left SMA and left superior cerebellum were negatively correlated with the changes of ACT scores before and after GCBT (*r* = −0.509, *P* = 0.044, Figure 5A). Meanwhile, one factor of CSHAI, the changes of NC scores, also showed negatively correlations with the changes of FC values between the right MTG and left superior cerebellum (*r* = −0.505, *P* = 0.046, Figure 5B).

### Therapeutic Effect Prediction

**FIGURE 6:**
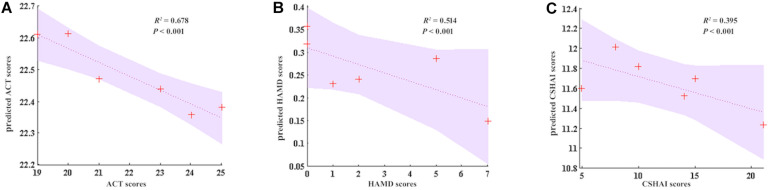
GCBT response prediction of asthma. **(A)** Five discriminant FC could accurately predict ACT (*R*^2^ = 0.678, *P* < 0.001). **(B)** Five discriminant FCs predict the 51.4% of HAMD (*R*^2^ = 0.395, *P* < 0.001). **(C)** Five discriminant FCs predict the 39.5% of CSHAI (*R*^2^ = 0.395, *P* < 0.001). ACT, asthma control test; HAMD, Hamilton depression rating scale; CSHAI, Chinese version of Short Health Anxiety Inventory.

In the current study, the therapeutic effect of GCBT was also predicted with linear SVR. We used the five discriminant connections of post-GCBT patients to predict their asthma control level, depression, and health anxiety. We found that our regression model using these five discriminant connections could predict the asthma control level after GCBT (*R*^2^ = 0.678, *P* < 0.001, Figure 6A). Moreover, they would also be used to predict the depression severity (*R*^2^ = 0.514, *P* < 0.001, Figure 6B) and health anxiety severity (*R*^2^ = 0.395, *P* < 0.001, Figure 6C).

## Discussion

It is the first study to identify therapeutic mechanism of GCBT in asthma using brain functional connections derived from resting-state fMRI. The current study further supported our previous findings that GCBT would play the therapeutic role by regulating abnormal brain activities (Zhang et al., 2017a,b). In the current study, we discovered discriminant FC patterns with the data-driven method including lasso, SVC, and *t*-test. These features would be the neuroimaging biomarkers of GCBT in asthma, which showed significant correlations with the clinical symptoms after treatment. And the improvement of asthma control level reduced by GCBT was significantly correlated with the changes of the FC between the left SMA and left superior cerebellum. Furthermore, these discriminant connections could predict individual patients’ asthma control level, and depressive and health anxiety symptoms after 8 weeks of GCBT. Thus, this discriminant brain connectivity presented advantages in its prognostic value for treatment response. Furthermore, they have high implications for understanding the therapeutic mechanisms of GCBT in asthma, as well as for identifying who will benefit most from GCBT.

Previous studies showed decreased regional cerebral blood flow and abnormal degree centrality in the right cerebellum of asthmatic patients compared with controls, which were consistent with our current findings (Li et al., 2018; Zhang et al., 2018). However, the relationships between abnormal brain activity and clinical symptoms were not found in those studies. The role of cerebellum in asthma has been further validated in the current study. We found that FC between superior cerebellum and other brain regions was associated with asthma, as well as associated with the improvement of emotional symptoms reduced by GCBT. Previous experiments demonstrate that the cerebellum can control several vegetative functions because it is connected with the limbic system (Strata, 2015). Meanwhile, the vegetative nervous system is associated with the maintenance of life, including breathing (Strata, 2015). It is thus plausible that GCBT would regulate breathing through autonomic nervous system to improve the asthma-related symptoms and then improve the asthma control level. Indeed, one prospective study has found that GCBT alleviated the chronic hyperventilation of patients with panic disorder (Beria et al., 2018). In the current study, both the right SFGmed and right MTG showed connection with the superior cerebellum. And these connections were increased after treatment close to the values of controls, as well as correlated with the asthma control level. These findings are consistent with those in patients with asthma (Zhang et al., 2017a; Li et al., 2018; Ritz et al., 2019) and chronic obstructive pulmonary disease (Zhang et al., 2013), which reported that the abnormal activity in the SFG and FC between right MTG and insula were both associated with lower asthma control level and stronger airway inflammation or arterial blood PO_2_. However, GCBT reversed the abnormal FC patterns between the superior cerebellum and SFG and MTG. It is thus plausible that our results may reflect the potential therapeutic mechanisms of GCBT in asthma.

Post-treatment connectivity between the superior cerebellum and SMA was also associated with depressive severity. This could reflect the potential link between emotion processing and motor circuitry in asthma. For example, a study in conversion disorder found increased FC in patients between the left amygdala and SMA (Hassa et al., 2017). Actually, breath is a complex motor function that needs the cooperation of neural activation and the skeletal muscles (Fogarty et al., 2018). The movement onset was often time locked to the frequent activity in SMA (Matsuzaka et al., 1992). The above findings seem likely to explain the FC changes induced by GCBT for asthma. Therefore, we speculated that GCBT might regulate the activity in SMA to improve breathing of asthmatic patients.

Cerebellar activation was revealed to be associated with the emotional processing (Stoodley and Schmahmann, 2009; Van Overwalle et al., 2014). Specifically, distinct sub-regions of the cerebellum are selectively involved in different primary emotions, including positive and negative emotions (Adamaszek et al., 2017). In addition, as a cortical hub, the insula carries the information of dyspnea and emotions (Borsook et al., 2016), and FC between the cerebellum and insula could play an important role in depression in asthmatic patients (Zhong et al., 2017; Samara et al., 2018). Specifically, in our study, this FC pattern was reversed by GCBT and positively correlated with depressive severity in patients who received treatment. Consistent with previous studies that abnormal FC would be recovered by GCBT in psychosis (Reggente et al., 2018; Tolmeijer et al., 2018), the current investigation also provided us a new insight to determine if the FC would be a neuroimaging biomarker of GCBT in improving depression in asthmatic patients.

Both the IFG and thalamus are associated with anxiety. For example, in patients with general anxiety disorder, FC between the IFG and precentral cortex were found to be significantly increased than in controls (Ma et al., 2019). It would be explained that IFG is one of vital structures involved in the processing of anxiety, and it intimately connects with the amygdala (Etkin et al., 2011). Thereby, it plays a critical role in emotional regulation. Moreover, the thalamus is a critical region involved in sensory information (Wijesinghe et al., 2015). Asthmatic patients pay more attention to their somatosensation, and they exaggerate their worry and panic, which may lead to different behaviors (Kew et al., 2016). So we speculated that the aberrant activity in thalamus was associated with anxiety induced by excessive attention to body sensation. The significant correlation between the FC and health anxiety in the current study just confirmed our speculation. Because GCBT can encourage individuals to challenge their unhelpful thoughts and form more realistic asthma-related sensation (Kew et al., 2016), the improvement of health anxiety may be associated with the recovery of FC values between the IFG and thalamus after GCBT.

For psychiatric disorders, classification accuracies based on fMRI data have been reported in range of 73–78.6% for first-episode drug-naïve schizophrenia (Mikolas et al., 2016; Cao et al., 2018), from 85 to 59% in adult autism spectrum disorder (Retico et al., 2016; Yahata et al., 2016), or 79% in panic disorder (Lueken et al., 2015). Compared with the studies of disease classification, almost all focus on psychiatric disease. Our finding provided the evidence that fMRI character could be a promising biomarker to differentiate asthmatic patients before and after GCBT. Beyond that, our findings could predict response of GCBT on the individual level. Notably, it is highly desirable to identify potential non-responders of GCBT before treatment. Additional treatment options could be provided for the patients who are not likely to respond (Hahn et al., 2015). Using ML, we demonstrated that pretreatment connections were most predictive of endpoint asthma control level. And the current study adds to the evidence of the role of connectivity in GCBT response across disorders. This could reflect the potential of certain individuals’ brain functional characteristics to reorganize and to provide a neural instantiation for emotional recognition and modified behaviors taught during GCBT. In the study on treatment response to CBT, Reggente et al. (2018) observed an accuracy of 67% for predicting response in OCD patients. In a similar vein, Coughlin et al. (2018) found that fMRI characteristic could predict the treatment response to GCBT in cigarette smokers. Our finding with an accuracy rate of 67.8% is in the range of previous reports.

There were several limitations in our study. One limitation of the current study is the small sample size. It will influence the correlation coefficient. Therefore, the complexity of GCBT in asthma warrants larger sample sizes to explain the underpinnings more fully. Furthermore, a new method such as deep learning can be used to find the biomarkers involved in therapeutic effect of GCBT for asthma based on large sample size. Another limitation is that only 17 patients completed the GCBT treatment; future work is required to control expulsion rate preferably.

This study marks a success in detecting neural underpinnings of GCBT as well as in predicting response to GCBT for asthma on the individual level. We applied a data-driven method to search the neuroimaging biomarkers of GCBT for asthma, and we demonstrated the efficacy of FC in assessing psychotherapy. This work substantially facilitates personalized treatment strategy of GCBT for asthma; meanwhile, it helps to improve response rates by selecting appropriate treatment.

## Data Availability Statement

The raw data supporting the conclusions of this article will be made available by the authors, without undue reservation.

## Ethics Statement

The studies involving human participants were reviewed and approved by Ethics Committee of Zhongda Hospital. The patients/participants provided their written informed consent to participate in this study.

## Author Contributions

YZ and YuY collected the fMRI and clinical data. YZ and KM performed the analysis and wrote the manuscript. YiY helped with data collection and revised the manuscript. ZH contributed to the fMRI data analysis and discussion. YoY and DZ designed the experiments and contributed to the manuscript revision. All authors contributed to the article and approved the submitted version.

## Conflict of Interest

The authors declare that the research was conducted in the absence of any commercial or financial relationships that could be construed as a potential conflict of interest.
